# Urban warming reshapes the safe outdoor activity opportunity window: dual-metric evidence from Heat Risk Hours and PAO

**DOI:** 10.3389/fpubh.2026.1815690

**Published:** 2026-07-22

**Authors:** Yuan Man, Shifei Zhao, Kang Li

**Affiliations:** 1 Faculty of Education, Universiti Putra Malaysia (UPM), Serdang, Selangor, Malaysia; 2 China National Basketball Academy, Shandong Sport University, Jinan, Shandong, China; 3 Department of Sport for All, Dongshin University, Naju, Republic of Korea

**Keywords:** dual-pathway squeeze, Heat Risk Hours, late-season concentration, outdoor activity opportunity window, PAO rate, urban adaptation governance, urban heat stress, UTCI

## Abstract

**Background:**

Against the backdrop of climate warming and intensifying extreme heat, urban heat stress not only elevates health risks but also disrupts the continuity of health promotion and public service provision by compressing the Meteorological feasibility of time resources for safely conducting outdoor physical activity. However, prior research has largely focused on linear changes in single heat indicators, with limited efforts to characterize—within a unified and comparable framework—the dual-pathway process of hazardous exposure-time expansion and safe activity-window contraction. Existing studies also rarely provide systematic quantification and validation of intra–warm-season risk reallocation, such as increasing late-season concentration and shifts in average peak timing. To address these gaps, we propose an Outdoor activity opportunity window framework that shifts the object of analysis away from observed physical activity behaviors—which are difficult to obtain continuously over long periods—toward a reproducible measure of Meteorological feasibility of time resources.

**Methods:**

Using Universal Thermal Climate Index (UTCI) statistics for 31 provincial capital cities and municipalities in China from 2010 to 2024, we developed a dual-metric system comprising Heat Risk Hours (hours under high-risk heat-stress conditions) and Physical Activity Opportunity (PAO) Rate (the share of time within the safe activity window). We applied robust trend estimation and standardized composite metrics to quantify the rate of deterioration in the opportunity window and to produce a tiered ranking of cities. We further conducted an external consistency validation of September UTCI risk hours using station-based observations of daily maximum temperature hot days (≥33°C/≥35°C). In addition, monthly data from May to September were used to test intra–warm-season risk reallocation and average peak timing: at the national scale, the late-season peak share (August–September) increased over time (≥33°C: 0.0076/year; ≥35°C: 0.0055/year), and the mean timing of peak risk shifted later overall (≥33°C: 0.0096 months/year; ≥35°C: 0.0071 months/year). In this study, PAO refers to the meteorology-constrained feasibility opportunity for outdoor physical activity and is intended to represent constraints on time resources; it is not equivalent to the amount of activity that actually occurs, nor is PAO Rate derived from survey-based participation data.

**Results:**

Our results show that: (1) a pronounced Dual-pathway squeeze exists at the national scale, with rising heat-risk exposure and shrinking safe activity windows occurring in parallel; (2) cities exhibit substantial heterogeneity in the “speed of worsening,” forming an identifiable tiered structure; and (3) warm-season risk undergoes structural reallocation along the time axis, with many cities showing signs of greater late-season concentration, although city-specific trajectories remain heterogeneous. As an application-oriented translation, we incorporated the rate of change in the share of the population aged 65 years and older as a dynamic vulnerability indicator, and coupled opportunity-window deterioration with aging dynamics to identify priority scenario cities. We quantified the magnitude of between-group differences using Cliff's delta (δ), a distribution-free effect size. Priority scenario cities displayed systematic differences in late-season structural indicators, suggesting that the temporal focus and resource prioritization of adaptation-oriented governance can be further refined.

**Conclusion:**

This study offers a reproducible dual-metric diagnostic framework linking urban heat risk and the Outdoor activity opportunity window, enabling the identification of priority contexts and supporting targeted investments in age-friendly cooling infrastructure, indoor substitute activity spaces, and late–warm-season warning–scheduling management for more precise resource deployment.

## 1 Introduction

Against the backdrop of sustained warming and intensifying extreme heat, urban heat stress has become a major climate-health risk affecting both population well-being and the functioning of urban systems (1–3). In the Chinese policy context, the “dual carbon” goals further underscore the urgency of aligning urban climate adaptation with broader low-carbon transition agendas (4). Hot and humid conditions not only increase the risk of heat-related illness, but also compress the feasible time periods during which residents can safely engage in outdoor physical activity, commuting, and other routine exertion. For urban governance, the central challenge is therefore not only how hot cities become, but also how high-risk periods expand across daily and seasonal time scales and reshape the time resources on which residents and public services depend (5–8).

Existing evidence on the heat–activity–adaptation nexus has grown rapidly. Heat-stress indices such as WBGT and UTCI are widely used to characterize human thermal load and thermal risk, while urban climate studies have documented changes in heatwave intensity, duration, and frequency (1–3, 9–11). Related work has also begun to estimate suitable activity hours or days from meteorological conditions, showing that heat can constrain health-promotion programming through time availability rather than through exposure alone (1–3). At the same time, vulnerability research highlights that environmental risk interacts with population structure, making equitable adaptation and resilience enhancement central concerns in city governance (1, 9–12).

However, three limitations remain. First, most studies still rely on a single heat metric and therefore do not jointly capture two governance-relevant processes: the expansion of hazardous exposure time and the contraction of the safe outdoor activity window (1–3). Second, city managers often need comparable indicators of the “speed of worsening” in order to rank cities, identify priority contexts, and allocate adaptation resources, yet such integrative and transparent metrics remain limited (1, 2, 5, 13). Third, relatively few studies track whether heat-related risk is becoming more concentrated in the later warm season, which would have direct implications for warning calendars, service continuity, and activity scheduling (1, 2, 5, 13).

To address these gaps, we adopt an Outdoor activity opportunity window perspective that shifts the analytical target away from observed physical activity behavior—which is difficult to collect continuously over long periods and to compare consistently across cities—toward a reproducible measure of meteorological feasibility of time resources. This perspective is intended to support city-scale diagnostics rather than causal inference on realized behavior. Recent interpretable machine-learning and geovisualization approaches have also provided complementary pathways for identifying nonlinear thresholds and heterogeneous effects under climate-related environmental change (14, 15). However, the present study prioritizes a transparent city-comparative framework that can be readily reproduced and translated into management practice.

Using data for 31 provincial capital cities and municipalities in China from 2010 to 2024, this study addresses four research questions. RQ1 asks whether opportunity windows exhibit a dual-pathway squeeze, namely rising heat-risk exposure alongside shrinking safe windows, and whether this pattern varies across cities. RQ2 asks whether cities can be stratified by the speed of worsening using a transparent composite trend indicator. RQ3 asks whether warm-season risk shows a relative shift toward the later part of the warm season, including greater concentration in August–September. RQ4 asks whether coupling opportunity-window deterioration with population aging dynamics can help identify priority governance scenarios with distinct late-season structural characteristics.

The study therefore aims to provide a reproducible diagnostic framework linking heat risk, activity opportunity, and dynamic vulnerability, while generating evidence that is interpretable for tiered urban adaptation and climate–health decision-making.

## 2 Data and measures

### 2.1 Study setting and spatiotemporal scope

This study examines 31 provincial capital cities and municipalities in China over the period 2010–2024 (16–18). China provides a policy-relevant setting for this analysis because it spans diverse climatic zones, contains very large urban populations, and faces concurrent pressures from rapid urbanization, climate warming, and growing demands for heat-health adaptation. These features make it especially suitable for testing a comparable city-level diagnostic framework intended to inform differentiated adaptation planning. Provincial capitals and municipalities were selected because they offer relatively consistent city-level data availability, occupy important positions in regional governance, and provide a comparable basis for cross-city diagnosis. The analytical units are the city–year level and the city–year–month level, with a warm-season focus on May–September (1, 16–18).

### 2.2 UTCI-based opportunity-window data and annual/monthly table structures

The opportunity-window system is derived from UTCI-based summary statistics generated from gridded near-surface thermal-stress products and harmonized to the city level (9–11, 16–18). UTCI was selected because it is a human-biometeorological index designed to characterize thermal stress under near-surface atmospheric conditions and is therefore more directly aligned with outdoor heat-stress experience than land-surface temperature products alone (10, 16–18). For each city, we constructed two linked data structures. The city–year table contains Heat Risk Hours, PAO Rate, Mean UTCI, Sum UTCI, and Total Hours. The city–year–month table for May–September records hours by UTCI risk tier (L1 Green, L2 Yellow, L3 Red, and L4 Purple) and derived indicators.

In this study, L1 Green and L2 Yellow represent the relatively safe or feasible portion of the activity window, whereas L3 Red and L4 Purple represent higher-risk thermal conditions. Accordingly, PAO hours are defined as L1 Green + L2 Yellow, Heat Risk Hours are defined as L3 Red + L4 Purple, and PAO Rate is defined as PAO hours divided by Total Hours (1, 2, 5, 13, 16–18). PAO refers to a meteorology-constrained opportunity for outdoor activity and should not be interpreted as realized activity behavior. Importantly, no survey-based sports-participation-rate dataset was used in constructing PAO Rate; instead, the indicator was calculated entirely from UTCI-tiered feasible hours.

### 2.3 External consistency validation data (station-based Tmax hot days)

To assess observational consistency, we used station observations of daily maximum temperature (*T*_max_) to construct monthly hot-day indicators: HotDays 33 ($T_{\max}\geq 33^{•}$C) and HotDays 35 ($T_{\max}\geq 35^{•}$C). These indicators were aggregated to the city level and compared with September UTCI-based risk hours (9, 10, 16–18). We used station-based near-surface air-temperature observations because the purpose of the validation was to test consistency with human-relevant atmospheric heat conditions measured at approximately 2 m above ground. This differs from land-surface temperature (LST), which is useful for mapping surface thermal patterns but does not directly represent the atmospheric thermal environment used in UTCI-based heat-stress assessment (19). The validation was therefore designed as a cross-source consistency check rather than a substitution of one thermal metric for another.

### 2.4 Core outcome measures: dual-pathway opportunity-window indicators

The two primary indicators are Heat Risk Hours (hours; risk pathway) and PAO Rate (0–1; opportunity pathway) (16–18). For each city, long-term rates of change over 2010–2024 were estimated for both indicators. To summarize the joint pace of worsening in a comparable way, we constructed a composite worsening index. The city-level outcome definitions and analytical variables are summarized in Table 1, and the composite worsening index is defined in Equation 1:


$$\begin{array}{l} W_{\text{index}}=Z\left(Risk\;slope\right)+Z\left(-PAO\;slope\right) \end{array}$$ (1)

where *Risk slope* is the city-specific trend in Heat Risk Hours, *PAO slope* is the city-specific trend in PAO Rate, and *Z*(·) denotes cross-city standardization. The minus sign on *PAOslope* aligns directionality so that larger values consistently indicate faster worsening: rising heat-risk exposure together with a more rapid contraction of the safe opportunity window.

**Table 1:** Key variables and operational definitions (16–18).

Variable	Definition	Unit	Resolution	Use in this study
Heat Risk Hours	Annual total hours classified as high heat-stress risk (UTCI risk tiers)	hours/year	City–year (2010–2024)	Primary “hazard-time” metric (risk-time expansion)
PAO rate	Share of hours within the physically-active opportunity window (safe/feasible UTCI tiers)	proportion (0–1)	City–year (2010–2024)	Primary “safety-time” metric (opportunity-window contraction)
Mean UTCI	Annual mean UTCI (background thermal stress intensity)	°C (UTCI)	City–year (2010–2024)	Context metric; supports interpretation and robustness
W index	Composite worsening-speed index: *Z*(Heat Risk Hours slope)+*Z*(−PAO Rate slope)	z-score	City (trend over 2010–2024)	Cross-city ranking and tiering of worsening speed (Figure 3D)
SepPeak33_share	Share of years with peak month in September (May–Sep window) for HotDays ≥33°C	proportion (0–1)	City (2010–2024 summary)	Season-structure shift; “September creep” indicator (HotDays33)
SepPeak35_share	Share of years with peak month in September (May–Sep window) for HotDays ≥35°C	proportion (0–1)	City (2010–2024 summary)	Season-structure shift; “September creep” indicator (HotDays35)
LatePeak_AugSep_Share_33	Share of peak-month occurring in Aug–Sep for HotDays ≥33°C (May–Sep window)	proportion (0–1)	City (2010–2024 summary)	Late-season peak shift (HotDays33)
LatePeak_AugSep_Share_35	Share of peak-month occurring in Aug–Sep for HotDays ≥35°C (May–Sep window)	proportion (0–1)	City (2010–2024 summary)	Late-season peak shift (HotDays35)
PeakMonth_33 / PeakMonth_35	Peak month (May–Sep) of monthly HotDays ≥33/35°C each year	month index (5–9)	City–year (May—Sep)	Average peak timing diagnostics (Figure 4B)
HotDays 33 / HotDays 35	Monthly count of days with station Tmax ≥33°C / ≥35°C	days/month	City–year–month	External physical validation & seasonal diagnostics
Age65 share_percent	Population share aged 65+	percent (%)	City–year (2010–2024)	Vulnerability / sensitivity proxy
Age65 slope_pp_per_year	Trend slope of Age65 share_percent over 2010–2024	percentage points/year	City (trend over 2010–2024)	Dynamic vulnerability; used for “double trap” identification
Quadrant	Four-category tiering by W index (≥0) × Z(Age65 slope) (≥0)	category	City	Scenario-based segmentation (Figure 2)

### 2.5 Seasonal-structure migration measures: late-season concentration and peak-month shift (May–September)

Within the May–September window, we defined three sets of structural indicators (1, 2, 5, 13, 16–18). First, LatePeak_AugSep_Share_33 and LatePeak_AugSep_Share_35 denote the proportion of years in which the peak month occurs in August–September. Second, SepPeak33_share and SepPeak35_share denote the proportion of years in which the peak month is specifically September. Third, PeakMonth_33 and PeakMonth_35 denote the peak month within May–September (month index 5–9), together with their drift rates in months per year. These measures were designed to capture whether risk becomes relatively more concentrated in the later part of the warm season.

### 2.6 Dynamic vulnerability measures: aging level and rate of change

**Figure 1:**
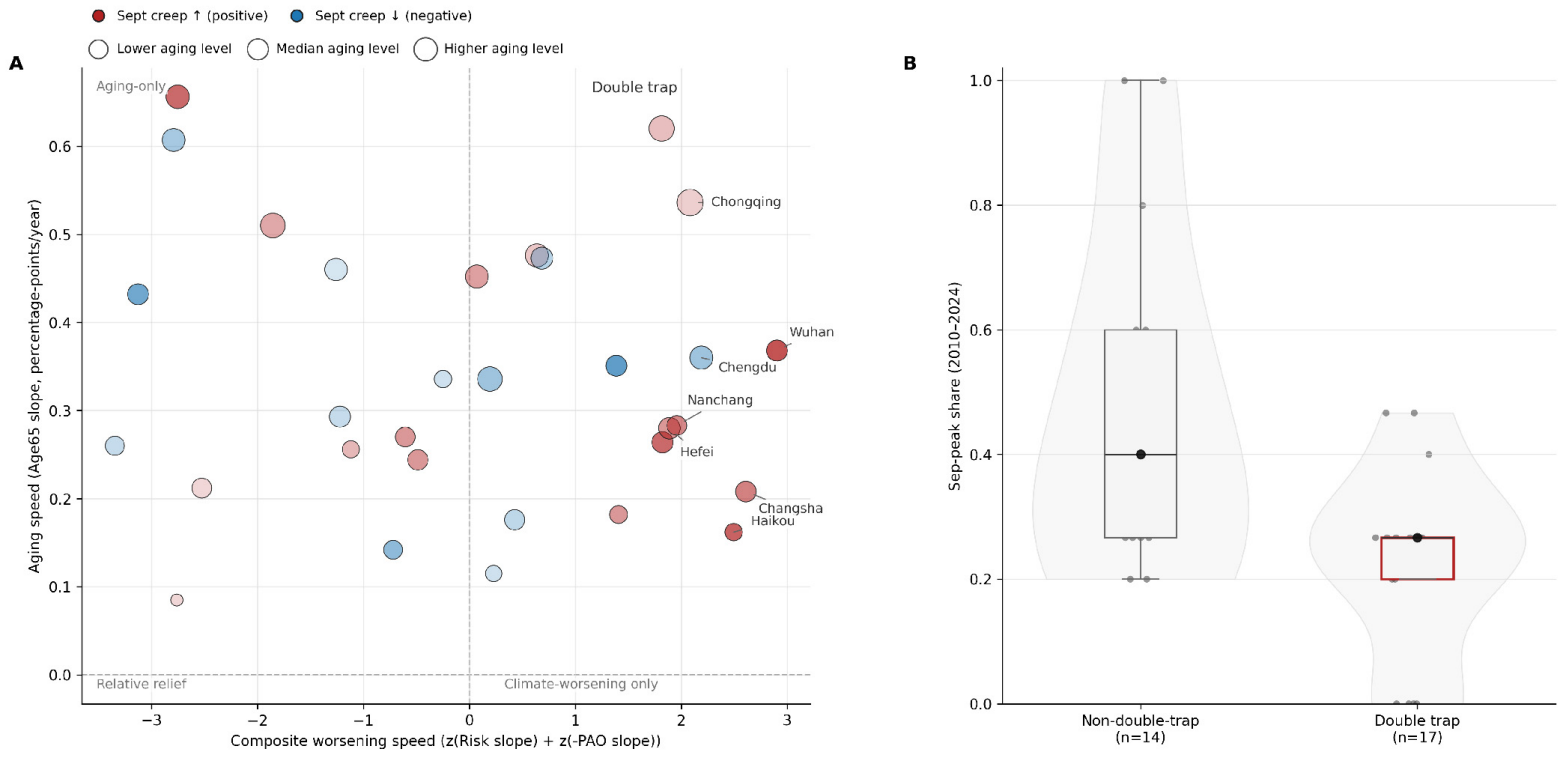
Priority scenario identification by coupling opportunity-window worsening speed with aging dynamics (27, 28). **(A)** A scenario space constructed using the integrated worsening speed (*W*_index_) and the pace of aging (Age65 slope), used to classify cities into scenario groups (27–29). **(B)** Differences in key late-season structural indicators between “Double trap” scenario cities and non–“Double trap” scenario cities (illustrated using Sep-peak share), showing a consistent differentiation signal linking “pressure scenarios” to late-season structure.

**Figure 2:**
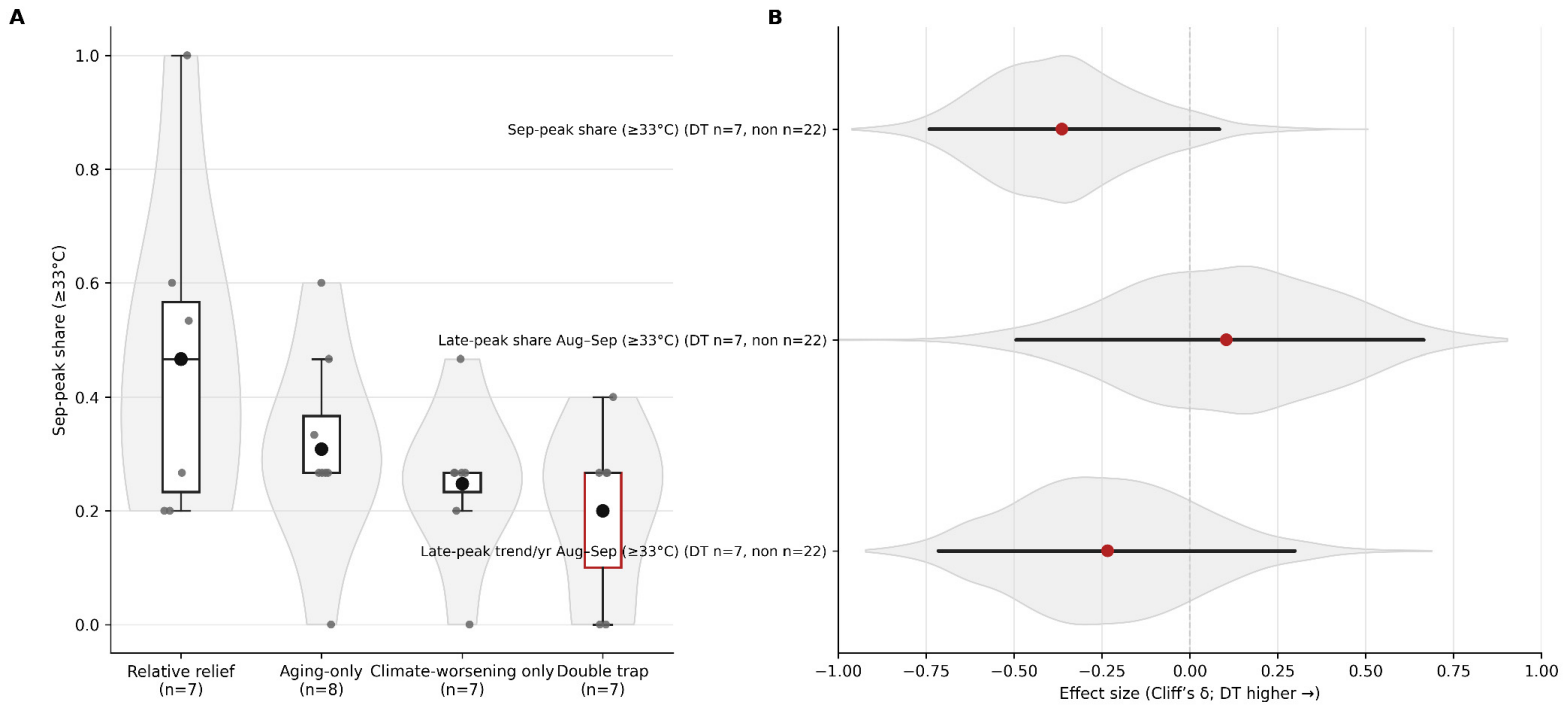
Four-quadrant tiered governance matrix and effect-size quantification (2, 20, 25, 26, 31, 36). **(A)** A four-quadrant tiered governance framework that combines the speed of change in adaptation pressure (*W*_index_) with dynamic sensitivity in population structure (Age65 slope), yielding differentiated capacity-building pathways (27–29). **(B)** Cliff's delta (δ), a distribution-free effect size, is used to quantify the magnitude of differences in key late-season structural indicators between “Double trap” scenario cities and non–“Double trap” scenario cities: Sep-peak share (≥33°C) δ = −0.529; Late-peak share (Aug–Sep) (≥33°C) δ = −0.080; Late-peak trend/year (Aug—Sep) (≥33°C) δ = 0.218 (20, 25–29). Cliff's δ is a distribution-free effect size. δ>0 indicates higher values in “Double trap” scenario cities, whereas δ < 0 indicates lower values in “Double trap” scenario cities (20, 25, 26).

To represent vulnerability dynamics, we used Age65 share_percent (%) and Age65 slope_pp_per_year (percentage points/year), which respectively capture the level and pace of population aging at the city level. These indicators were compiled from official city-level demographic statistics in the harmonized city-year dataset used for this study and were retained because aging is a policy-relevant population characteristic closely linked to heat vulnerability in the public-health literature (5, 6, 12). In the analytical framework, aging is treated as a contextual vulnerability indicator rather than a causal determinant of opportunity-window change. It is coupled with *W*_index_ to identify priority scenario cities (Figures 1, 2) (1, 5, 6, 12, 16–18).

## 3 Methods

### 3.1 Overall framework and inferential boundaries

We constructed a descriptive evidence chain—long-term trends → warm-season structural change → overlay of dynamic vulnerability—to evaluate changes in the time resources represented by the opportunity window. The unit of assessment is the meteorological feasibility of time resources, not realized physical activity behavior or directly observed health outcomes. Accordingly, the study is designed as a city-comparative diagnostic framework and does not make causal claims regarding behavior or health effects (1, 5, 6, 10, 12, 20–22). This inferential boundary is important because the framework is intended to provide reproducible, policy-oriented diagnosis of environmental time-resource constraints rather than to replace individual-level behavioral or causal models. Risk-tier definitions and thresholds follow commonly used UTCI classification schemes. In Supplementary material, we further report sensitivity comparisons using alternative composite-weighting schemes and alternative trend-estimation approaches to examine the stability of the main conclusions (Supplementary Tables S4, S5) (9, 10, 20–22).

### 3.2 City-level long-term trend estimation (Theil–Sen)

For each city, we estimated long-term rates of change in Heat Risk Hours and PAO Rate over 2010–2024 (10, 20–22). Trend slopes were computed using the Theil–Sen estimator, with units of h/year for Heat Risk Hours and proportion/year for PAO Rate (18, 21–24). The Theil–Sen estimator was selected because it is robust to outliers, performs well for monotonic trend estimation in relatively short annual series, and is widely used in environmental time-series analysis where year-to-year variability may be substantial.

### 3.3 Integrated worsening index (worsening index)

To synthesize the joint pace of deterioration in a transparent and comparable way, we defined the integrated worsening index for city *i*. The city-specific implementation of the integrated worsening index is shown in Equation 2:


$$\begin{array}{l} W_{i}=Z\left(s_{risk,i}\right)-Z\left(s_{pao,i}\right) \end{array}$$ (2)

where *s*_*risk, i*_ = *slope*(*HeatRiskHours*) (h/year), *s*_*pao, i*_ = *slope*(*PAORate*) (proportion/year), and *Z*(·) denotes cross-city standardization (10, 20–22). Cross-city standardization was used to place the two trend components on a comparable scale before combination. Because a more negative PAO trend reflects faster window contraction, subtracting *Z*(*s*_*pao, i*_) aligns the two components so that larger *W*_*i*_ values consistently indicate a faster rise in heat-risk exposure together with a faster loss of safe opportunity time (1, 2, 5, 10, 13, 20–22).

This index is intended as a transparent composite trend indicator for ranking and stratification rather than as a mechanistic interaction model. Its purpose is to summarize, on a comparable city-level scale, whether the risk pathway and the opportunity pathway are worsening together. We therefore use it as a decision-support index for cross-city diagnosis, not as evidence that the two underlying processes are statistically independent or causally interacting in any specific parametric form. To further test its stability, Supplementary material report (i) a ranking comparison based on PCA-derived weights for *W*_index_ and (ii) an alternative trend-estimation approach using OLS with heteroskedasticity-robust standard errors (HC3). These checks evaluate whether weighting choices or slope-estimation methods materially alter the core city rankings and tiered conclusions (Supplementary Tables S4, S5) (1, 2, 5, 10, 13, 20–22).

### 3.4 External consistency validation (September UTCI risk hours vs. station hot days)

We used Spearman's rank correlation to assess monotonic consistency between September UTCI risk hours and station-based hot-day indicators, including HotDays 33 (≥33°C) and HotDays 35 (≥35°C) (10, 20–22). Spearman correlation was chosen because the purpose of this analysis was to test ranked agreement in observational intensity rather than to impose a linear functional form. City-specific validation results are summarized in Supplementary Table S3 (9, 10, 20–22).

### 3.5 Testing warm-season structural migration (May–September)

Using monthly data from May through September, we examined whether warm-season risk became relatively more concentrated in the later part of the season (1, 2, 5, 13). Three indicators were analyzed. First, PeakMonth (6–9, 13) captures the annual peak-risk month for each city within May–September and its drift rate (months/year). Second, LatePeak share (Aug–Sep) captures the share of years in which the peak month occurs in August–September and its trend (proportion/year). Third, Sep-peak share captures the share of years in which the peak month is September and its trend (proportion/year) (1, 2, 5, 10, 13, 20–22). Complete city-level results are reported in Supplementary Table S2 (1, 2, 5, 10, 13, 20–22).

### 3.6 Priority scenario identification and effect size (Cliff's δ)

We used *W*_*i*_ to represent the speed of change in adaptation pressure and Age65 slope to represent the pace of population-aging dynamics. Cities were classified into four quadrant-based scenarios using the thresholds *W*_*i*_≥0 and *Z*(*Age*65*slope*)≥0 (Figure 1A) (10, 20–22). This scenario typology was designed as an interpretable decision-support rule rather than as an unsupervised clustering procedure. In this framework, the “Double trap” scenario denotes cities with both above-average worsening speed and above-average aging dynamics, while the remaining quadrants capture climate-pressure dominant, aging-sensitive, and relatively mitigated contexts. Between-group differences were quantified using Cliff's delta (δ), a distribution-free effect size (Figure 2B) (5, 6, 10, 12, 20–22, 25, 26).

## 4 Results

### 4.1 National-scale dual-pathway squeeze: concurrent risk expansion and window contraction

**Figure 3:**
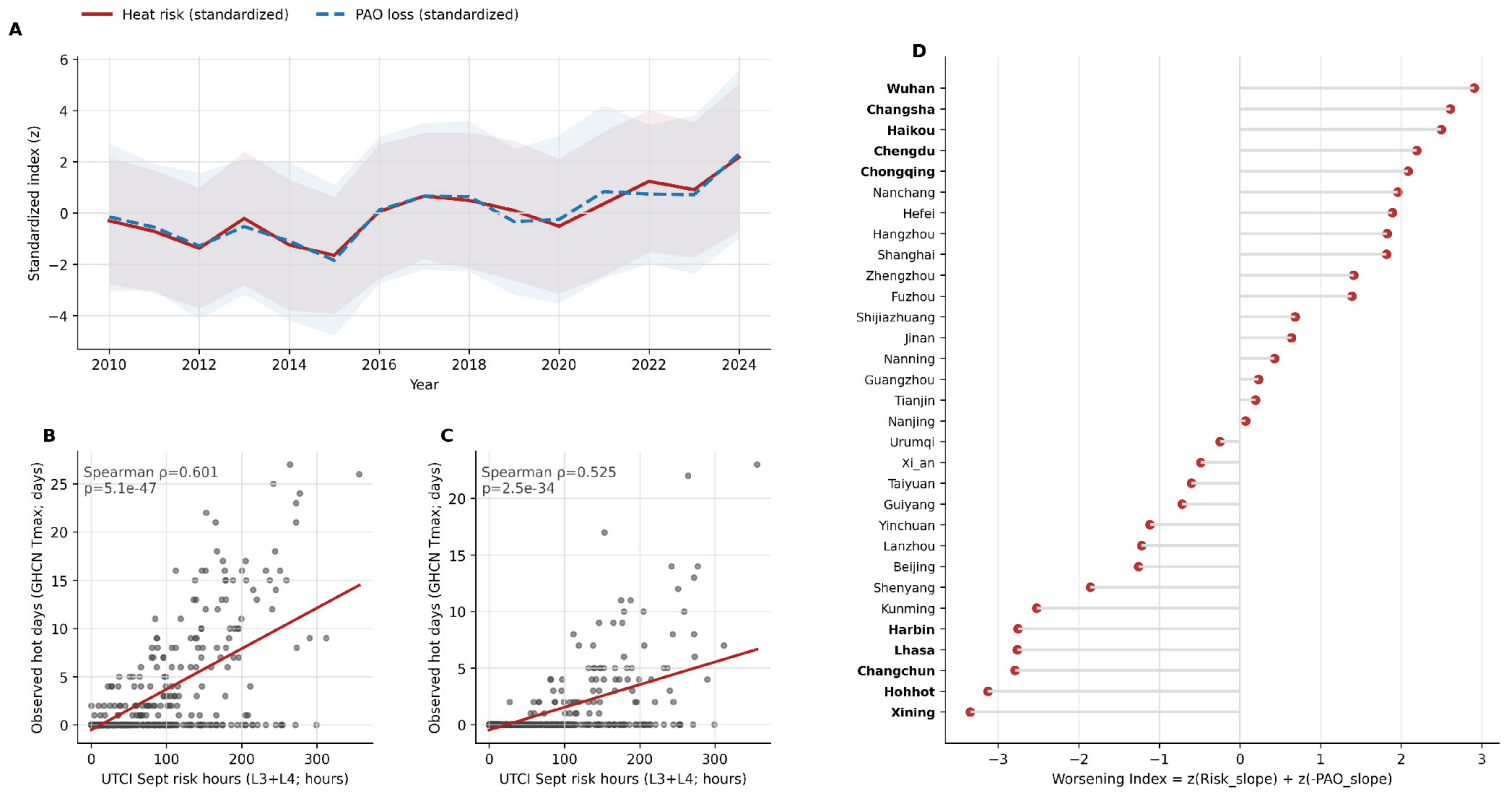
National-scale Dual-pathway squeeze, external consistency validation, and tiered city stratification by worsening speed (2010–2024). **(A)** Standardized series averaged across the 31 cities: Heat Risk Hours increases in parallel with declines in PAO Rate, indicating a clear dual-metric squeeze pattern in the Outdoor activity opportunity window (27–29). **(B, C)** Consistency validation between September UTCI risk hours and station-based Tmax hot days (≥33°C/≥35°C): Spearman ρ = 0.601, *p* = 5.06 × 10^−47^; ρ = 0.525, *p* = 2.53 × 10^−34^ (10, 27–29). **(D)** The integrated worsening index *W*_index_ (= Z(Risk slope) + Z(-PAO slope)) is used to support cross-city ranking and tiered stratification (27–29). PAO Rate denotes the share of time within the safe activity window. “PAO loss” refers to the downward trend in PAO Rate; to align directions, -PAO slope is included in *W*_index_ (27, 28). HotDays 33/35 denote station-based counts of Tmax hot days (≥33°C/≥35°C), and WarmNights 25/28 denote station-based counts of Tmin warm nights (≥25°C/≥28°C). *n* is the number of available city–year observations. Statistical testing is based on Spearman's rank correlation (24).

At the national scale over 2010–2024, Heat Risk Hours increased steadily while PAO Rate declined during the same period, indicating a clear dual-pathway squeeze: the amount of hazardous exposure time expanded while the safe and feasible Outdoor activity opportunity window contracted (Figure 3A) (27–29). The two national trend lines should therefore be interpreted as concurrent worsening in the risk and opportunity dimensions, rather than as evidence of perfectly symmetric or mechanically opposite trajectories. On a long-term time scale, these patterns indicate increasing constraints on the time resources available for safe outdoor physical activity.

In addition, station-based extreme temperature observations and the UTCI dual-metric system showed significant consistency both for the September window and under pooled warm-season specifications (Table 2; Supplementary Table S3), providing external credibility support for the subsequent findings on inter-city differentiation and seasonal structural migration (10, 27–30).

**Table 2:** External consistency validation results at the warm-season pooled scale (May–September).

Pair	*n*	Spearman ρ	*p*-value
Heat Risk Hours vs. HotDays 33	403	0.537	1.81 × 10^−31^
Heat Risk Hours vs. HotDays 35	403	0.566	1.43 × 10^−35^
PAO rate vs. WarmNights 25	403	–0.803	2.46 × 10^−92^
PAO rate vs. WarmNights 28	403	–0.496	2.05 × 10^−26^

UTCI tier labels (L1–L4) follow the study's tiering scheme (10, 17, 18).

### 4.2 Heterogeneity in the speed of worsening across cities: the integrated worsening index reveals tiered stratification

At the city scale, the “speed of worsening” shows pronounced heterogeneity (27–29). After ranking cities using the integrated worsening index *W*_index_, cities with faster deterioration cluster into a tier characterized by both more rapid growth in heat-risk exposure and faster contraction of the safe opportunity window. In contrast, another group exhibits lower *W*_index_ values, indicating a slower overall pace of deterioration (Figure 3D). This stratification provides an operational quantitative handle for setting priorities in tiered governance and capacity building (Table 3).

**Table 3:** Core results (selected cities reported in the main text; 2010–2024).

W_rank	City	Heat_Risk _Hours	Risk _slope	PAO _Rate	PAO _slope	W index	Age65 _share	Age65 _slope	Quadrant
1	Wuhan	1096.2	22.20	0.359	–0.0048	2.904	10.71	0.368	Double trap
2	Changsha	1062.5	15.86	0.366	–0.0071	2.610	10.49	0.208	Climate-pressure
3	Haikou	1123.1	17.40	0.135	–0.0060	2.495	8.21	0.162	Climate-pressure
4	Chengdu	733.8	13.33	0.542	–0.0072	2.191	12.54	0.360	Double trap
5	Chongqing	1020.0	13.85	0.395	–0.0066	2.084	15.47	0.536	Double trap
6	Nanchang	1077.5	15.08	0.330	–0.0057	1.959	9.69	0.283	Climate-pressure
7	Hefei	878.6	16.00	0.432	–0.0050	1.889	11.15	0.280	Climate-pressure
8	Hangzhou	937.8	16.18	0.402	–0.0048	1.824	10.87	0.264	Climate-pressure
9	Shanghai	548.4	11.73	0.522	–0.0069	1.816	14.44	0.620	Double trap
10	Zhengzhou	836.9	12.25	0.504	–0.0055	1.408	8.43	0.182	Climate-pressure
11	Fuzhou	937.1	12.00	0.320	–0.0056	1.387	10.67	0.351	Double trap
12	Shijiazhuang	883.5	12.60	0.491	–0.0033	0.685	11.44	0.473	Double trap
13	Jinan	787.8	12.00	0.503	–0.0035	0.638	12.64	0.476	Double trap
16	Tianjin	713.5	10.50	0.514	–0.0030	0.194	13.74	0.336	Double trap
17	Nanjing	816.3	6.80	0.460	–0.0045	0.071	12.34	0.452	Double trap
22	Yinchuan	370.5	9.60	0.582	0.0002	–1.118	8.04	0.256	Lower-pressure
23	Lanzhou	306.9	8.75	0.612	0.0001	–1.221	10.82	0.293	Lower-pressure
24	Beijing	790.1	5.00	0.502	–0.0016	–1.258	11.92	0.460	Aging-sensitive
25	Shenyang	321.1	6.17	0.590	0.0006	–1.855	13.94	0.510	Aging-sensitive
26	Kunming	2.5	0.22	0.859	–0.0004	–2.525	9.85	0.212	Lower-pressure
27	Harbin	187.1	0.67	0.599	0.0004	–2.753	12.68	0.656	Aging-sensitive
28	Lhasa	0.0	0.00	0.481	0.0001	–2.761	5.29	0.085	Lower-pressure
29	Changchun	184.0	2.36	0.606	0.0014	–2.791	12.33	0.607	Aging-sensitive
30	Hohhot	82.9	1.00	0.582	0.0016	–3.127	10.66	0.432	Aging-sensitive
31	Xining	0.0	0.00	0.552	0.0018	–3.346	9.34	0.260	Lower-pressure

Larger W index values indicate faster increases in risk alongside faster declines in the safe window. Heat Risk Hours_mean is the 2010–2024 mean number of high-risk hours (h). Risk slope is the long-term rate of change in Heat Risk Hours (h/year). PAO slope is the rate of change in PAO Rate (/year), where negative values indicate window contraction (21–23). Full results for all 31 cities are provided in Supplementary Table S1.

### 4.3 Seasonal structural migration: greater late-season concentration and later average peak timing

**Figure 4:**
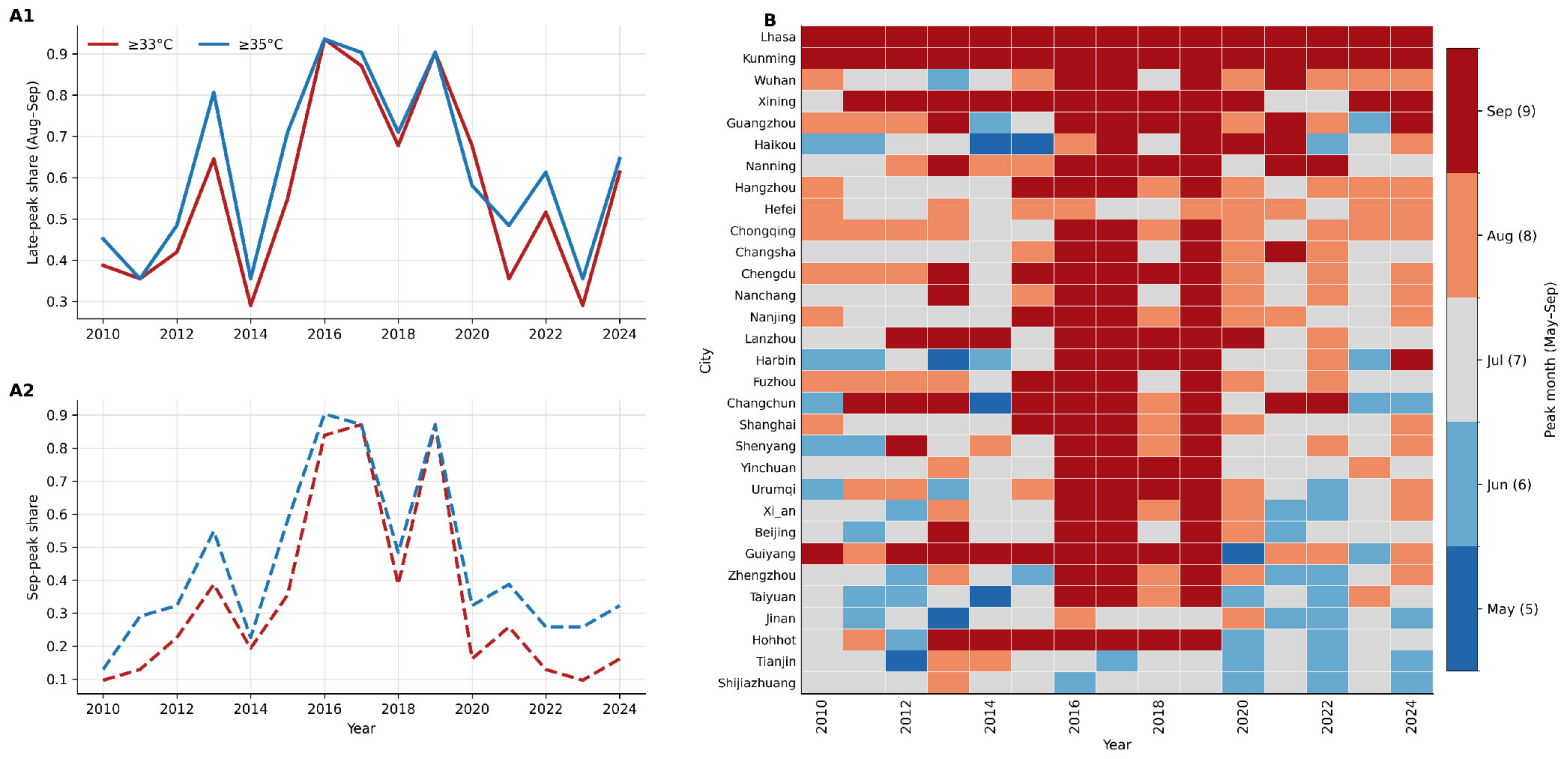
Evidence of May–Sep structural migration (2010–2024): greater late-season concentration and later average peak timing (30, 37). (**A1, A2)** National-level time series of the late-season peak share (August–September) and the September-peak share, reported under two threshold specifications (≥33°C and ≥35°C) (27–29). **(B)** City–year heatmap of the peak month (May–September), illustrating cross-city heterogeneity in peak timing and its temporal redistribution. The “peak month” is defined as, for each city and each year, the month within May–September (month index 5–9) with the highest risk intensity (17, 18).

Beyond long-term trends, warm-season structural indicators suggest that heat risk is being reallocated along the time axis, with increasing concentration toward the latter part of the warm season (27–29). At the national scale, the share of years in which the peak month falls in August–September increased over time (≥33°C: 0.0076/year; ≥35°C: 0.0055/year). In parallel, the average timing of the peak month shifted later overall (≥33°C: 0.0096 months/year; ≥35°C: 0.0071 months/year). Taken together, these indicators are consistent with greater late-season concentration at the national level, although city-specific trajectories remain heterogeneous rather than uniform (Figure 4). For some cities, the relevant structural indicators show clearer late-season strengthening, whereas others show weak, mixed, or even negative slopes in parts of the indicator set (Table 4).

**Table 4:** Seasonal structural migration indicators (selected cities reported in the main text; May—Sep) (16–18, 30, 37).

City	Quadrant	LatePeak33 (Aug-Sep)	Slope LatePeak33	LatePeak35 (Aug–Sep)	Slope LatePeak35	SepPeak33	SepPeak35	Slope Peak33	Slope Peak35
Wuhan	Double trap	0.667	0.0607	0.733	0.0464	0.267	0.400	0.0929	0.0393
Haikou	Climate-pressure	0.400	0.0536	0.400	0.0536	0.267	0.267	0.1500	0.1821
Hangzhou	Climate-pressure	0.667	0.0500	0.667	0.0143	0.267	0.333	0.0464	0.0143
Harbin	Aging-sensitive	0.400	0.0500	0.667	0.0429	0.333	0.667	0.1464	0.1321
Changsha	Climate-pressure	0.467	0.0393	0.533	0.0143	0.267	0.333	0.0571	0.0250
Nanjing	Double trap	0.600	0.0250	0.667	0.0179	0.267	0.267	0.0214	0.0143
Shanghai	Double trap	0.533	0.0107	0.600	0.0143	0.267	0.267	0.0071	0.0071
Jinan	Double trap	0.133	0.0071	0.267	–0.0143	0.000	0.067	0.0000	-0.0393
Chongqing	Double trap	0.800	0.0036	0.733	0.0179	0.200	0.200	0.0071	0.0214
Shijiazhuang	Double trap	0.067	–0.0143	0.000	0.0000	0.000	0.000	–0.0643	–0.0679
Chengdu	Double trap	0.800	–0.0250	0.667	0.0250	0.400	0.400	–0.0393	0.0321
Tianjin	Double trap	0.133	–0.0250	0.133	–0.0214	0.000	0.000	–0.0429	–0.0929
Guiyang	Lower-pressure	0.867	–0.0321	1.000	0.0000	0.600	1.000	–0.1429	0.0000
Hohhot	Aging-sensitive	0.533	–0.0464	0.733	–0.0286	0.467	0.733	–0.0821	–0.0857
Fuzhou	Double trap	0.667	–0.0536	0.467	–0.0250	0.267	0.267	–0.0571	–0.0286

LatePeak denotes the share of years in which the peak month falls in August—September; SepPeak denotes the share of years in which the peak month is September. PeakMonth_slope is the drift rate of the peak month (5–9) over time (months/year) (21–23). Full results for all 31 cities are provided in Supplementary Table S2.

### 4.4 Priority scenario identification: coupling opportunity-window worsening speed with aging dynamics

After coupling *W*_index_ (the speed of change in adaptation pressure) with Age65 slope (dynamic sensitivity in population structure), the 31 cities separate into four scenario groups in the scenario space: the “Double trap” scenario (high worsening × high aging), climate-pressure dominant, aging-sensitive, and relatively mitigated (Figure 1A) (27–29). Cities in the “Double trap” scenario imply that, while the opportunity window is deteriorating at an accelerated pace, the share of vulnerable populations is also rising more rapidly. This combination suggests that priorities for maintaining public service continuity and strengthening adaptive capacity should be advanced and targeted earlier in these settings.

### 4.5 A four-quadrant tiered governance matrix and effect-size quantification

Building on the scenario identification, we further provide a four-quadrant tiered governance matrix and quantify the magnitude of between-group differences in key late-season structural indicators using Cliff's delta (δ), a distribution-free effect size (Figure 2) (27–29). The results indicate that “Double trap” scenario cities and non–“Double trap” scenario cities exhibit identifiable differences in several late-season structural indicators. These quantified contrasts offer interpretable support for (i) time-priority decisions (e.g., whether management windows should be extended into the late warm season) and (ii) resource prioritization (e.g., which scenarios warrant earlier, front-loaded investments in capacity building) (20, 25–29).

### 4.6 External consistency validation (warm-season city–year pooled): Stable agreement between indicators and station-based extreme temperature observations

**Figure 5:**
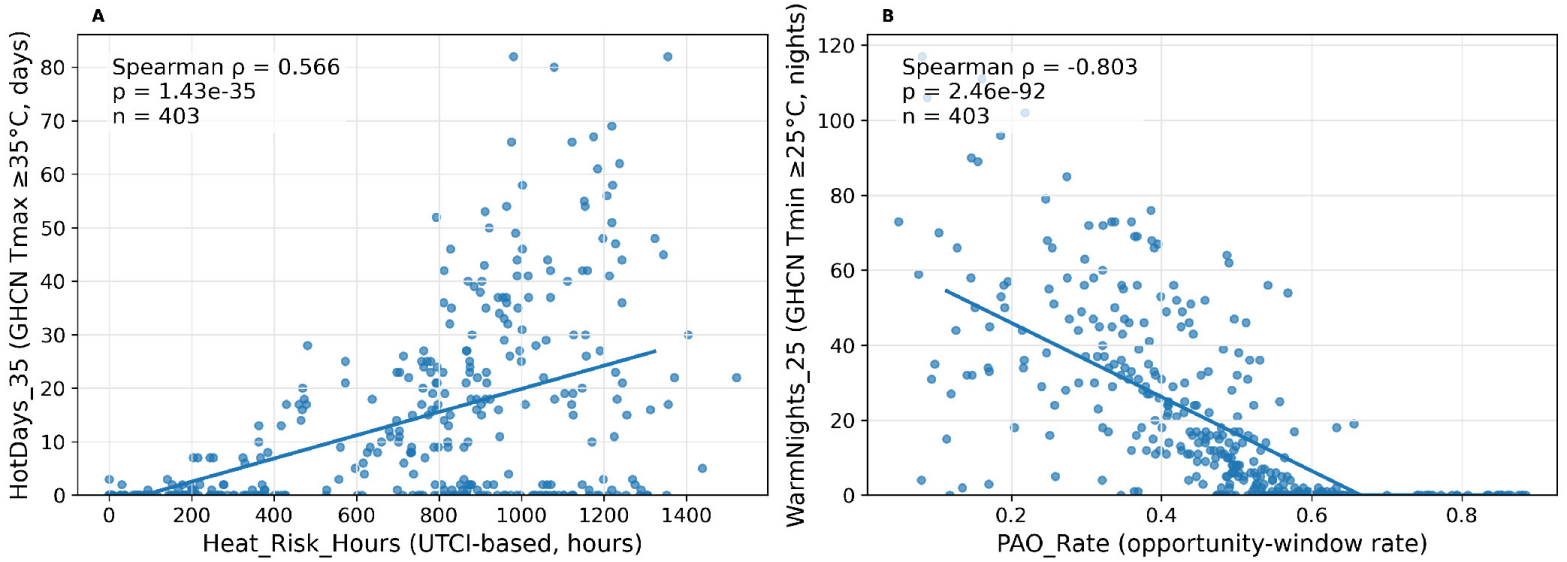
Warm-season external consistency validation: Agreement between city–year indicators and station-based extreme temperature observations (2010—2024; May–Sep) (9, 10, 16–18, 30, 37). **(A)** Association between Heat Risk Hours (UTCI-based, hours) and HotDays 35 (GHCN Tmax ≥35°C, days): Spearman ρ = 0.566, *p* = 1.43 × 10^−35^, *n* = 403 (10, 27–29). **(B)** Association between PAO Rate (opportunity-window rate) and WarmNights 25 (GHCN Tmin ≥25°C, nights): Spearman ρ = −0.803, *p* = 2.46 × 10^−92^, *n* = 403 (27–29). *n* denotes the number of matched city–year records. The fitted line is shown for visual guidance only; statistical inference is based on Spearman's monotonic association (24).

Building on the September-window validation (Figures 3B, C; Table 2; Supplementary Table S3), we further tested the consistency between the dual indicators and station-based extreme temperature observations at the warm-season city–year pooled scale (May–September) (27–29). The results show that Heat Risk Hours is significantly and positively correlated with hot-day counts, whereas PAO Rate is significantly and negatively correlated with warm-night counts (Figure 5). This pattern indicates that the dual-metric opportunity-window system retains stable external consistency over a broader temporal aggregation, providing credibility support for subsequent city stratification and structural migration analyses.

Using matched city–year observations (*n* = 403), Heat Risk Hours shows significant positive monotonic associations with station-based extreme hot-day counts: Spearman ρ = 0.537 (*p* = 1.81 × 10^−31^) with HotDays 33 (Tmax ≥33°C) and Spearman ρ = 0.566 (*p* = 1.43 × 10^−35^) with HotDays 35 (Tmax ≥35°C). In parallel, PAO Rate is significantly negatively associated with warm-night frequency: Spearman ρ = −0.803 (*p* = 2.46 × 10^−92^) with WarmNights 25 (Tmin ≥25°C) and Spearman ρ = −0.496 (*p* = 2.05 × 10^−26^) with WarmNights 28 (Tmin ≥28°C). Overall, these external validation results support the robustness and physical interpretability of the proposed risk–opportunity-window framework from the standpoint of observational consistency.

## 5 Discussion

### 5.1 Resilience-oriented interpretation of the main findings

From an Outdoor activity opportunity window perspective, this study provides dual-pathway evidence that urban heat risk constrains the time resources underlying health promotion: Heat Risk Hours increased (hazardous exposure-time expansion) while PAO Rate decreased (safe feasibility-window contraction) over the same period from 2010 to 2024, forming a national-scale dual-metric squeeze (5, 12, 31). At the city level, the integrated worsening speed (*W*_index_) reveals substantial heterogeneity in the “speed of worsening.” At the seasonal-structure level, the evidence is more appropriately interpreted as greater late-season concentration and later average peak timing at the national scale, rather than as a perfectly uniform delay across all cities. Compared with conventional descriptions that simply report “hotter” or “more frequent” heat events, these findings map more directly onto key management questions in urban resilience governance: when hazardous exposure time lengthens while safe windows shrink, public sports services, community health-promotion programming, and the operation of public spaces require more precise time-slot strategies and stronger continuity safeguards (2, 5, 12, 31, 32).

### 5.2 Plausible mechanisms and an interpretive framework

The dual-metric squeeze can be understood as a time-domain projection of an intensified heat-stress background: the synchronous rise in high-risk hours and decline in feasible-window share reflects concurrent changes in hazardous exposure and feasible time resources (5, 12, 31). The observed late-season concentration and later average peak timing indicate structural adjustments in the within-season boundary and timing of warm-season risk, which may be associated with interacting factors such as regional background warming, compounded heat–humidity conditions, urban heat-island effects, and constraints on nighttime heat dissipation. We do not conduct causal identification; however, this interpretive framework supports an actionable governance proposition: the temporal focus of adaptation should not be limited to a single midsummer peak, but should extend to sustained monitoring and response into the late warm season where the structural indicators suggest growing pressure (2, 5, 12, 31).

### 5.3 Tiered governance and capacity building: differentiated pathways from an urban resilience perspective

Based on the evidence chain of risk expansion → window contraction → greater late-season concentration → dynamic population structure, we suggest establishing tiered governance pathways centered on the speed of change in adaptation pressure (*W*_index_) and dynamic sensitivity in population structure (Age65 slope) (Table 5) (5, 12, 31).

**Table 5:** Tiered governance and decision-support matrix (four-quadrant scenarios).

Quadrant	Identification rule	Governance focus	Timing focus	Priority actions (examples)	Suggested KPIs
Double trap	W index ≥0 AND Z(Age65 slope) ≥0	Resilience capacity upgrade + service continuity for vulnerable groups	Extend management from midsummer to late warm-season (Aug–Sep)	Age-friendly cooling micro-environments; indoor alternatives; community cooling/hydration network; late-season scheduling & warning–service linkage	Heat Risk Hours slope ↓; PAO Rate slope ↑; SepPeak35_share ↓; Age-friendly facility coverage ↑
Climate-pressure	W index ≥0 AND Z(Age65 slope) < 0	Hazard-driven adaptation and peak-load management	Prioritize hottest periods + monitor late-season creep	Heat alert refinement; cooling of public spaces; demand management for outdoor events; shaded corridors	Heat Risk Hours slope ↓; PAO Rate slope ↑; peak-month shift monitored
Aging-sensitive	W index < 0 AND Z(Age65 slope) ≥0	Vulnerability-oriented service access and support	Focus on safe-hour access across the warm season	Targeted outreach; barrier-free access to safe indoor venues; tailored guidance for older adults	PAO Rate level ↑; participation access indicators; emergency response readiness
Lower-pressure	W index < 0 AND Z(Age65 slope) < 0	Preventive monitoring and maintenance	Early detection of emerging late-season signals	Routine monitoring; maintain cooling infrastructure; contingency planning	Stability of Heat Risk Hours & PAO Rate; early warning indicators

This matrix translates the coupling of the speed of change in adaptation pressure (*W*_index_) and dynamic sensitivity in population structure (Age65 slope) into actionable governance priorities, time-priority targets, and example action lists, facilitating tiered allocation and continuous monitoring under budget constraints (2, 31, 38).

For cities in the “Double trap” scenario, priorities may include strengthening continuity and accessibility of public spaces and public services under high-heat conditions—such as age-friendly cooling micro-environments, indoor substitute activity spaces, and community cooling-and-hydration support networks—while extending management focus from midsummer into the late warm season to respond to late-season concentration-related time-slot pressures. For climate-pressure dominant cities, emphasis can be placed on refining risk monitoring, early-warning thresholds, and time-slot management, alongside public-space cooling measures and service surge capacity to reduce peak impacts. For aging-sensitive cities, strategies may focus on improving adaptive support and service accessibility for vulnerable groups, strengthening community-level health-promotion services and care networks. For relatively mitigated cities, the priority may be maintenance and preventive monitoring, with particular attention to early signals of risk intensification in the late warm season. This implication is also consistent with broader evidence that greener and more thermally moderated urban environments can contribute to health gains, including lower mortality risks, while also intersecting with patterns of social and material deprivation. Accordingly, adaptation-oriented improvements in shading, cooling micro-environments, and accessible public spaces may yield co-benefits that extend beyond thermal comfort alone (33, 34). Overall, *W*_index_ and the four-quadrant tiered matrix can serve as a complementary module in the urban resilience governance toolbox, supporting a shift from episodic response toward an optimized pathway of continuous monitoring → fine-grained management → capacity enhancement (2, 5, 12, 31, 32).

### 5.4 Limitations and future work

First, the object of assessment in this study is a reproducible, city-scale measure of the meteorological feasibility of time resources, designed to support long-term monitoring and tiered diagnostics. Its relationship with observed behavior may be moderated by adaptive adjustments, such as within-day time substitution, access to cooled indoor settings, and behavioral avoidance (5, 12, 31). Future work could integrate wearable-device data, location-based mobility traces, or usage records from public sports facilities to test, on consistent time scales, how well opportunity-window indicators explain shifts in realized activity timing (2, 5, 12, 31, 32).

Second, city-mean summaries may mask within-city spatial heterogeneity. Subsequent research could combine remotely sensed urban heat-island indicators with street-level microclimate measurements and facility accessibility data to enable zonal diagnostics, thereby improving the targeting of localized adaptation measures (2, 5, 12, 31, 32).

Third, the composite worsening index is designed as a transparent tool for comparison and prioritization rather than as a full causal or interaction model. Although supplementary analyses show that the main rankings are stable under alternative weighting and trend specifications, future studies could examine nonlinear thresholds and interaction structures in greater depth using richer covariate information and alternative modeling strategies. Future work could also integrate threshold detection, geovisualization, and interpretable modeling strategies to examine heterogeneous city-specific responses more explicitly (14, 15).

Fourth, beyond population aging, socioeconomic conditions, the share of outdoor workers, greening and shading conditions, and the accessibility of public spaces may also shape equity in time-resource availability. The proposed matrix is therefore intended as an extensible framework; where data permit, future studies could incorporate multidimensional vulnerability indicators to build a more comprehensive evidence base for tiered governance (5, 12, 29, 31, 35).

## 6 Conclusions

Using UTCI-based opportunity-window summaries for 31 provincial capital cities and municipalities in China from 2010 to 2024, this study proposes and validates an Outdoor activity opportunity window framework for city-scale diagnostics. We find that: (1) a national-scale dual-pathway squeeze is evident, with hazardous exposure-time expansion occurring in parallel with safe feasibility-window contraction, indicating sustained compression of time resources for safe outdoor activity over the long term; (2) cities diverge substantially in their “speed of worsening,” and the integrated worsening index (*W*_index_) provides an operational basis for cross-city ranking and priority setting in tiered governance; (3) warm-season risk undergoes structural reallocation, with evidence of greater late-season concentration and later average peak timing at the national scale, suggesting that the temporal focus of governance should extend beyond midsummer toward sustained management later in the warm season where local indicators warrant it; and (5) coupling opportunity-window deterioration with aging dynamics enables identification of priority cities in the “Double trap” scenario (high worsening × high aging), and scenario-based comparisons provide decision-relevant evidence for resource deployment and capacity building.

Overall, this study offers reproducible quantitative levers to support continuous monitoring, fine-grained management, and resilience capacity enhancement under budget constraints. The framework can inform prioritized deployment of age-friendly cooling infrastructure, indoor substitute activity spaces, and integrated late–warm-season early-warning and scheduling strategies.

## Data Availability

The original contributions presented in the study are included in the article/Supplementary material, further inquiries can be directed to the corresponding authors.
